# Impact assessment of Benazir Nashonuma Programme (BNP) on maternal, child health and nutritional status in Pakistan: a quasi-experimental study protocol

**DOI:** 10.1136/bmjopen-2024-094565

**Published:** 2025-06-27

**Authors:** Shah Muhammad, Asma Abdul Malik, Sajid Soofi, Atif Habib, Muhammad Umer, Arjumand Rizvi, Imran Ahmed, Jef Leroy, Simon Cousens, Zulfiqar Ahmed Bhutta

**Affiliations:** 1 CoE in Women and Child Health, The Aga Khan University, Karachi, Pakistan; 2 Pediatrics and Child Health, The Aga Khan University, Karachi, Pakistan; 3 International Food Policy Research Institute, Washington, UK; 4 Infectious and Tropical Diseases, London School of Hygiene and Tropical Medicine, London, UK; 5 SickKids Centre for Global Child Health (C-GCH), Toronto, Ontario, Canada

**Keywords:** NUTRITION & DIETETICS, Social Support, Surveys and Questionnaires, EPIDEMIOLOGY, Health, Community child health

## Abstract

**Introduction:**

Maternal and child malnutrition is a significant public health concern in Pakistan, with 40% of children under five being stunted. In response, the Government of Pakistan initiated the Benazir Nashonuma Programme (BNP), a nutritional supplementation programme for pregnant women, mothers of children aged 0 to 23 months and children aged 6 to 24 months. This study aims to evaluate the effectiveness of the BNP in reducing childhood stunting and improving maternal and child health outcomes.

**Methods and analysis:**

A quasi-experimental longitudinal study comprising baseline, midline and endline surveys will be conducted across 18 districts (9 intervention and 9 control) in Pakistan. The surveys will use a two-stage cluster sampling method to enrol 13 200 children aged 0–59 months and their mothers from the Benazir Income Support Programme households. The primary outcome of interest is the prevalence of under-five stunting. We will use a difference-in-differences approach to estimate the impact by comparing the documented changes over time between the intervention and control groups.

**Ethics and dissemination:**

This study will provide critical insights into the effectiveness of the BNP in addressing childhood undernutrition in Pakistan. The findings will inform policy and programmatic decisions aimed at reducing undernutrition in resource-constrained settings. Ethical approval has been obtained from the Ethics Review Committee of Aga Khan University and the Pakistan National Bioethics Committee.

**Trial registration number:**

NCT06025786.

**STRENGTHS AND LIMITATIONS OF THIS STUDY**

- The study covers 13 200 children under five across 18 districts using a two-stage cluster sampling method for comprehensive population coverage.
- The pre-intervention and post-intervention design with control groups allows for a rigorous evaluation of the impact of the Benazir Nashonuma Programme.
- The study measures various maternal and child health indicators, including nutritional status, dietary diversity and healthcare utilisation.
- Non-random assignment of intervention districts by the programme may introduce bias. There is a risk of spillover effects, where households in control areas, especially in districts bordering intervention districts, may indirectly benefit from the intervention, potentially diluting the measured impact. The study’s findings may not generalise to non-Benazir Income Support Programme-registered households or other vulnerable populations not enrolled in the programme. Self-reported data on outcomes like antenatal care and food security may introduce recall bias, and the assumption of parallel trends in the control and intervention groups may not always hold.

## Introduction

 Maternal and child undernutrition remains a pervasive public health challenge, particularly in low-income countries. Child undernutrition encompasses stunting, wasting, being underweight and micronutrient deficiencies, with significant regional variation.^1^ Undernutrition contributes to nearly half (45%) of all deaths among children under five^2^ and has been widely associated with compromised physical and cognitive development, with far-reaching consequences throughout an individual’s life.^3^

Stunting, characterised by suboptimal linear growth among children, arises from the persistent lack of adequate energy and nutrient intake needed for proper growth and development.^4^ South Asia bears the highest burden, with an estimated 57.9 million stunted children under five.^5^ In Pakistan, 40% of children under five are affected by stunting.^6 7^ The first 1000 days of a child’s life, from conception to the age of 2 years, represent a critical period that offers a unique window of opportunity for interventions to enhance maternal and child nutrition and health outcomes.^8^ The WHO antenatal care guidelines recommend balanced energy-protein (BEP) supplementation for pregnant women in undernourished populations to reduce the risk of stillbirth and the incidence of small-for-gestational-age newborns.^9 10^ Conditional cash transfers tied to the utilisation of specific services by pregnant women and lactating mothers have been found to reduce poverty and improve human development outcomes.^11 12^ A recent study in Sindh, Pakistan, has reported the benefits of lipid-based nutritional supplements, reducing the risk of stunting by 9% and wasting by 22% among children 0–24 months of age,^13^ while a randomised trial conducted in Rahim Yar Khan district of Punjab in 2019 reported a 14% reduction in under-two stunting, underscoring the effectiveness of lipid-based nutritional supplements coupled with cash transfers in reducing stunting among children 6–23 months of age in marginalised populations.^14^ A systematic review of studies conducted in low- and middle-income countries on the effectiveness of BEP supplements on birth outcomes reported a 60% reduction in stillbirth rates and a 40% decrease in low birth weight (LBW) among women who consumed the supplements.^15^ A recent two-stage meta-analysis of four randomised controlled trials demonstrated that small-quantity lipid-based nutrient supplements provided to pregnant women reduced the risk of low birth weight by 11%, stunting by 17% and wasting by 11% compared with iron and folic acid or standard care.^16^

### Intervention: Benazir Nashonuma Programme (BNP)

To address the alarming prevalence of stunting in Pakistan, the Benazir Income Support Programme (BISP) launched a nutrition-focused initiative, the BNP, in collaboration with the World Food Programme in August 2020. As the country’s largest social safety programme, BISP seeks to mitigate economic hardships by offering quarterly unconditional cash transfers amounting to 8500rupees (equivalent to US$30.4) to approximately 9 million vulnerable women residing in the poorest households.^17^

The National Socio-Economic Registry (NSER) is an ongoing national survey that identifies eligible BISP beneficiary households through the application of a Proxy Means Test (PMT)-based Poverty Score Card. The PMT score offers an objective way to estimate a household’s level of welfare and poverty using indicators associated with monetary welfare measures. The NSER is regularly updated to ensure that BISP effectively targets the poorest 20% of households, thereby maximising the programme’s impact on poverty alleviation.^18^ Payments are distributed through six partner banks, which leverage their branch networks, retail agents and franchises or collaborate with mobile phone companies to reach beneficiaries in remote areas.

**Figure 1:**
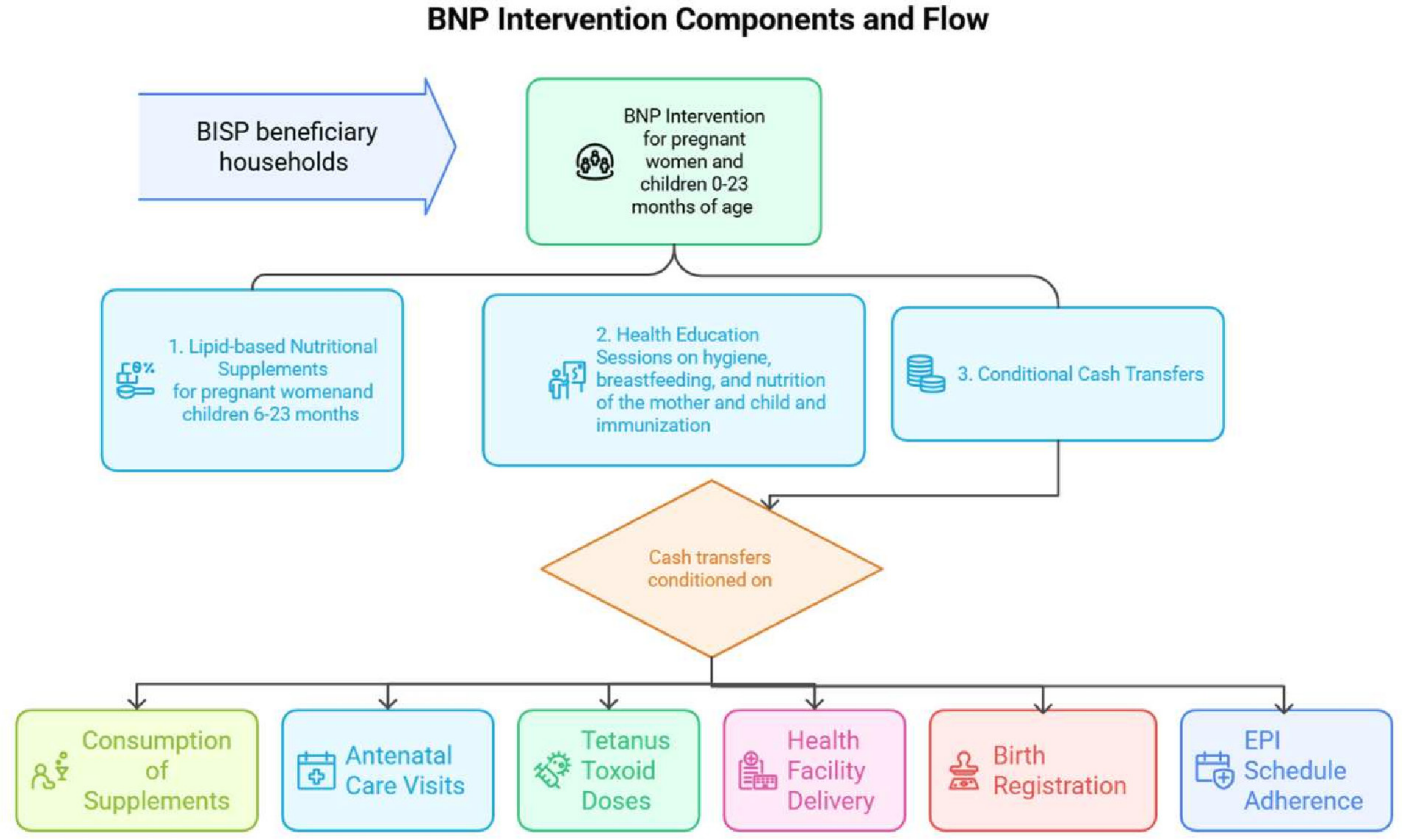
The flow of different intervention components.

The BNP operates as a health and nutrition initiative for pregnant and lactating women and children aged 6–24 months who are registered in the BISP.^19^ The BNP facilitation centres located in the subdistricts of intervention clusters are established within government health facilities, mostly in secondary healthcare facilities (District Headquarters and Tehsil Headquarters). These centres serve as the central point for delivering intervention and are integrated with the government’s health service delivery system. The programme is currently being operated in 552 facilitation centres in 157 districts of Pakistan, including 31 mobile centres in Sindh.^20^ Within this programme, the beneficiaries receive a quarterly supply of lipid-based nutrition supplements on their visit to the facilitation centres (FC) and a conditional cash transfer, contingent on the supplement’s consumption, tetanus vaccination, antenatal care attendance and participation in health and nutrition awareness sessions. FC workers verify the beneficiaries’ compliance with supplements and attendance at awareness sessions, after which they stamp the beneficiaries’ BNP cards to authorise the cash transfer. Then, the beneficiaries collect cash transfers from the nearest point-of-sale agents after biometric verification. Figure 1 demonstrates the flow of different intervention components.

The objective of this evaluation is to assess the impact of the BNP on reducing the prevalence of stunting among children aged 0–59 months of age and other key maternal and child health outcomes.

## Methods and analysis

### Study design and sites

This study will employ a quasi-experimental longitudinal design with data collection at baseline, midline and endline in 18 districts across all four provinces and two administrative areas of Pakistan (Pakistan is divided into four provinces: Khyber Pakhtunkhwa, Punjab, Sindh and Balochistan). The administrative areas of Azad Kashmir and Gilgit-Baltistan have their own respective political and administrative machinery. The provinces are further divided into divisions and districts.^21^ The study started in December 2021 and is expected to end in June 2026. This study aims to evaluate the impact of the Nashonuma Programme on child nutrition indicators—stunting, wasting and underweight among children aged 0–59 months—and underweight prevalence in women of reproductive age (15–49 years). It will assess reproductive health service utilisation, including antenatal, delivery and postnatal care, as well as minimum dietary diversity among women. Additionally, the study will examine the impact on infant and young child feeding (IYCF) practices, birth registration and immunisation coverage for children aged 0–59 months. Coverage and uptake of the BNP will be evaluated through enrolment rates and utilisation of nutrition supplementation and health services among pregnant women, lactating mothers and children aged 0–23 months. We hypothesise that BNP implementation will result in a 7.5% relative reduction in stunting prevalence among children aged 0–59 months over 3 years, decreasing from 48.2% to 44.6% in intervention districts compared with control districts without BNP.

We engaged the government from the outset in selecting both intervention and control districts. The government also informed us of the decision to scale up the intervention nationally and assisted in identifying suitable districts for inclusion in the assessment. While the government selected the most affected districts for the intervention, the research team identified control districts, ensuring they were matched to the intervention districts within the same province. The matching was done based on a comparative assessment of key indicators, including the prevalence of under-five stunting and the mean body mass index (BMI) among non-pregnant women of reproductive age from the National Nutritional Survey of Pakistan 2018.^22^ The selected intervention and control districts, along with the key indicators for the selection of these districts, are presented in table 1. The baseline survey will be conducted over 3 months. The endline survey will be conducted 3 years after the baseline survey.

**Table 1:** Key indicators for selection of the intervention and control districts for each province (National Nutritional Survey 2018)

Province/region	Intervention districts	Under-five child stunting prevalence (%)	Mean BMI among non-pregnant WRA (kg/m^2^)	Matched control districts	Under-five Child stunting prevalence (%)	Mean BMI among non-pregnant WRA (kg/m^2^)
Punjab	Rajanpur	48.4	22.6	Bahawalnagar	43.8	23.3
Sindh	Dadu	49.2	22.7	Naushero Feroze	52.8	22.1
	Shaheed Benazir Abad	54.8	22	Sangarh	50.7	21.9
Khyber Pakhtunkhwa (KP)	Upper Dir	46.3	24.6	Shangla	46.4	24.8
	Tank	50.7	25.7	I. Khan	49.2	24.3
Baluchistan	Lasbela	42.7	20.2	Sibbi	44.2	22.0
	Harnai	49.4	24	Sherani	53.4	22.7
Azad Jammu and Kashmir (AJK)	Bagh	46.5	24.3	Muzaffarabad	45.7	22.9
Gilgit Baltistan (GB)	Diamer	48.5	23.2	Shiger	51.8	22.4

### Eligibility criteria

All households in both the intervention and control districts that are registered in the BISP and have at least one child between the ages of 0–59 months will be eligible for inclusion in the study regardless of their enrolment status in the BNP. Including children 24 to 59 months will allow us to capture the impact of the intervention on children who were exposed to BNP before 24 months of age.

### Intervention: Benazir Nashonuma Programme (BNP)

BEP is a locally produced medium-quantity lipid-based nutritional supplement (LNS-MQ) created with heat-treated chickpeas, vegetable oils, dry skimmed milk, sugar, vitamins, minerals, emulsifiers and antioxidants.^14^ The supplement for pregnant and lactating women (Maamta) is a nutrient-dense supplement, with the majority of the (>50%) energy derived from lipids and less than 25% of total calories derived from protein.^23^ A 75-gram sachet is recommended for daily use and is known to improve maternal nutritional status among undernourished populations^23 24^ (table 2). The supplement for children 6–23 months of age (Wawamum) similarly provides the required nutrients mainly by lipids and includes energy, protein, micronutrients and essential fatty acids. A 50-gram dose of Wawamum covers the recommended daily intake of most micronutrients and provides 255 kcal of energy, approximately one-quarter of the daily energy requirements for this age group^13^ (table 3). These ready-to-use products are intended as supplements to the regular diet, do not require refrigeration and have a long shelf life.^25^ The cash transfers amount to 2500 Pakistani rupee (PKR) (US$8.86) per quarter for pregnant women until childbirth. After the child is born, the cash transfer increases to 3000 (US$10.63) PKR for a girl child, while remaining at 2500 PKR for a boy child.

**Table 2:** Composition of nutrient values in 75 grams LNS-PLW supplement (Maamta)

Nutrient	Amount	Nutrient	Amount
Energy	400 kcal	Vitamin E	12 mg
Protein	10.5 g	Vitamin K	20.2 mcg
Lipids	24 g	Vitamin C	45 mg
Sodium	<203 mg	Vitamin B1 (thiamine)	0.75 mg
Potassium	675 mg	Vitamin B2 (riboflavin)	1.57 mg
Calcium	400 mg	Vitamin B3 (niacin)	9.75 mg
Phosphorus	337 mg	Vitamin B5 (pantothenic acid)	3 mg
Magnesium	112 mg	Vitamin B6	1.35 mg
Iron	7.5 mg	Vitamin B7 (biotin)	45 μg
Zinc	8.2 mg	Vitamin B9 (folates)	247 μg
Copper	1.0 mg	Vitamin B12	2.0 μg
Selenium	15 μg	Dry skimmed milk protein	2.7 g
Iodine	75 μg	Fatty acid n-6	1.95 g
Manganese	0.9 mg	Fatty acid n-3	0.22 g
Vitamin A	412 μg	Fibre content	<5%
Vitamin D	11.2 μg	Moisture	<2.50%

LNS-PLW, a lipid-based nutritional supplement for pregnant and lactating women.

**Table 3:** Composition of nutrient values in 50 grams LNS-MQ supplement for 6–23 months children (Wawamum)

Nutrient	Amount	Nutrient	Amount
Energy	255 kcal	Vitamin E	8 mg
Protein	5.5 g	Vitamin K	13.5 μg
Lipids	13 g	Vitamin C	30 mg
Sodium	<135 mg	Vitamin B1 (thiamine)	0.5 mg
Potassium	450 mg	Vitamin B2 (riboflavin)	1.05 mg
Calcium	267 mg	Vitamin B3 (niacin)	6.5 mg
Phosphorus	225 mg	Vitamin B5 (pantothenic acid)	2.0 mg
Magnesium	75 mg	Vitamin B6	0.9 mg
Iron	5 mg	Vitamin B7 (biotin)	30 μg
Zinc	5.5 mg	Vitamin B9 (folates)	165 μg
Copper	0.7 mg	Vitamin B12	1.35 μg
Selenium	10 μg	Dry skimmed milk protein	1.8 g
Iodine	50 μg	Fatty acid n-6	1.3 g
Manganese	0.6 mg	Fatty acid n-3	0.15 g
Vitamin A	275 μg	Fibre content	5.0%
Vitamin D	7.5 μg	Moisture	<2.50%

LNS-MQ, a lipid-based nutritional supplement medium quantity; LNS-PLW, a lipid-based nutritional supplement for pregnant and lactating women.

To receive cash benefits, participants must meet a set of conditionalities, including providing evidence of consuming at least 90% of the nutritional supplement allocated for the quarter, substantiated by presenting the empty sachets. Additionally, they must attend three antenatal care visits throughout pregnancy, receive two doses of tetanus toxoid and participate in a total of 11 health and nutrition education sessions during pregnancy and the first 23 months of the child’s life. Three of these sessions must occur during pregnancy and eight in the postpartum period. These sessions cover important topics such as hygiene, breastfeeding and nutrition for the mother and child. Other conditionalities include delivering in a health facility, ensuring birth registration and adhering to the recommended Expanded Programme on Immunisation schedule for the child.

### Outcomes and variables

The primary outcome of this study is stunting prevalence in children 0 to 59 months of age. Stunting is defined as a height-for-age z-score below −2 using the WHO growth standards for children aged 0–59 months.^26^ The secondary outcomes encompass several maternal and child health indicators (table 4). Demographic and household characteristics of study participants will also be recorded, including socioeconomic status, assistance recipient’s status and food insecurity

**Table 4:** List of primary and secondary outcomes for BNP quasi-experimental study

Outcome	Definition
Primary outcome	
Change in the prevalence of stunting among children 0–59 months of age	Percentage of children with a height-for-age z-score below −2 SD using the WHO growth standards for children aged 0–59 months.
Secondary outcomes	
Change in the prevalence of non-pregnant underweight women of reproductive age (15–49 years)	Percentage of non-pregnant WRA with a body mass index (BMI) less than 18.5 kg/m^2^.
Change in the prevalence of wasting among children 0–59 months of age	Percentage of children with weight-for-height z-score below −2 SD using the WHO growth standards for children aged 0–59 months.
Change in the prevalence of acute malnutrition using MUAC among children 0–59 months of age	Percentage of children 0–59 months of age with a mid-upper-arm circumference (MUAC) less than 12.5 cm.
Change in the number of antenatal care (ANC) visits during pregnancy	Number of antenatal care (ANC) visits done during the preceding pregnancy, leading to a live birth within the last 2 years.
Change in the prevalence of institutional deliveries	Percentage of women who gave birth at a healthcare facility during the preceding pregnancy, leading to a live birth within the last 2 years.
Change in the prevalence of skilled birth attendance	Percentage of women whose birth was attended by skilled health personnel during the preceding pregnancy, leading to a live birth within the last 2 years.
Change in prevalence of utilisation of iron folic acid (IFA) during pregnancy	Percentage of WRA consuming IFA tablets during pregnancy.
Change in the prevalence of minimum dietary diversity for women (MDDW)	Percentage of WRA consuming at least five out of the ten food groups during the previous day and night (24 hours).^28^
Change in the prevalence of infants who were ever breastfed	Percentage of infants who were breastfed at least once.
Change in the prevalence of exclusive breastfeeding among infants 0–5 months of age	Percentage of infants who were fed exclusively with breast milk during the previous day.
Change in the prevalence of diarrhoea among children 0–59 months of age	Percentage of children 0–59 months of age who had diarrhoea in the last 4 weeks preceding the survey.
Change in the prevalence of food insecurity status at household level	Food Insecurity Experience Scale (FIES) by the Food and Agriculture Organisation (FAO) is an experience-based measure of household or individual food security. It consists of eight questions regarding people’s access to adequate food in the last 12 months due to lack of money or other resources. Food insecurity will be reported as mild, moderate and severe.^29^

### Sample size and sampling strategy

The sample size was calculated based on the prevalence of stunting among under-five children in the lowest two wealth quintiles, as reported in the National Nutritional Survey (NNS) of.^7 22^ BISP poverty survey data could not be used for this calculation as it did not include information on stunting and maternal nutrition, which are critical indicators for our study. At least 13 200 households (6600 in the intervention arm and 6600 in the control arm) will be required to detect a 7.5% relative reduction in the prevalence of stunting over a 3-year period, decreasing from 48.2% to 44.6%. This calculation assumes 90% power, a 5% level of significance, a 90% response rate and a design effect of 1.5. The sample size of 13 200 households will be evenly distributed across 18 districts, resulting in a required sample size of 734 households per district (for a total of 13 212 households).

**Figure 2:**
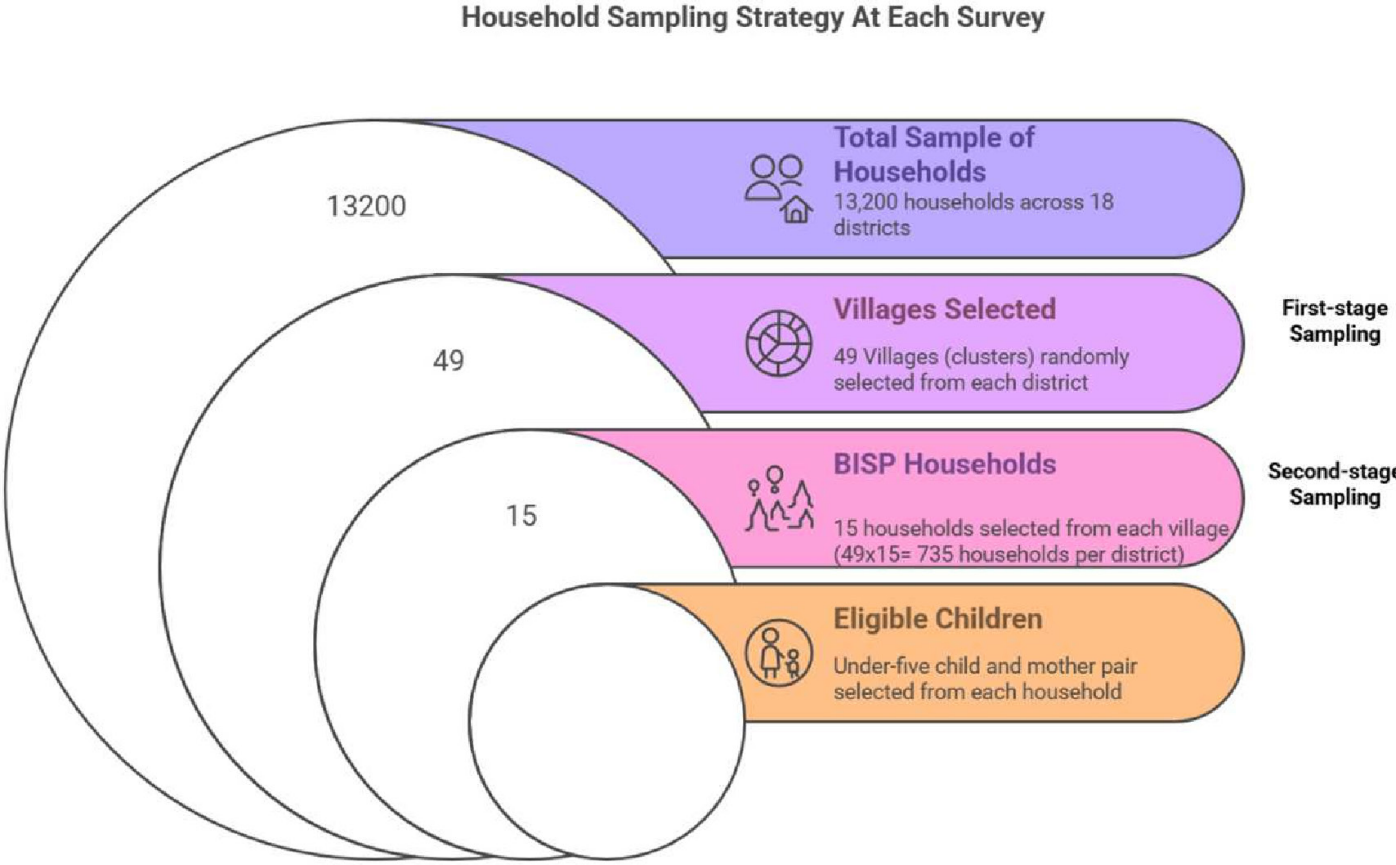
The cluster sampling strategy.

A two-stage cluster sampling strategy will be employed for study enrolment. In the first sampling stage, 49 villages per district will be randomly selected as primary sampling units (PSUs) by dividing the total sample of 735 households per district equally, allocating 15 households to each of the 49 clusters. In the second stage, all BISP registered households will be line listed, and 15 BISP households or secondary sampling units (SSUs) from each village that have at least one under-five child will be randomly selected from the list of BISP beneficiary households (49 villages (PSU) × 15 households (SSU) = 735 households per district). If a selected household has more than one under-five child, one child will be randomly chosen using the Kish grid method,^27^ along with their respective mother or caregiver. For each chosen household, a Kish grid table will be used to list all eligible children aged 0–59 months residing in the household. The children will be ranked in descending order of age. In instances of refusal to participate, the next child on the Kish grid will be selected. Figure 2 illustrates the cluster sampling strategy.

### Data collection

A structured questionnaire, drawing from questionnaires such as the Pakistan Demographic and Health Survey and Pakistan NNS 2018, has been developed for the surveys. The questionnaire was translated from English to Urdu and then back-translated to English to ensure accuracy. The Data Management Unit at the Aga Khan University (AKU) developed a Java-based application with SQLite programming to enable a Computer Assisted Personal Interview approach to data collection using electronic tablets, ensuring real-time validation and quality control checks to enhance data accuracy and completeness.

The data collection teams will be recruited locally by the core research team in each district. Each district will have five teams, each comprising two female interviewers and a district supervisor responsible for ensuring data quality and adherence to protocols. All data collectors will undergo extensive 2-week centralised training, which will include classroom sessions on survey methodology, the sampling frame and a question-by-question review of the questionnaire. Sessions on anthropometric measurements will be conducted following the SMART methodology. Interviewers will be evaluated through a standardisation test, provided with feedback and required to participate in mock surveys and field practice. The training will conclude with a 2-day pilot data collection. During fieldwork, data collection teams will identify selected households, obtain written informed consent from the selected woman of reproductive age and conduct interviews with pregnant women or mothers/caretakers of under-five children. In addition, anthropometric measurements will be taken of all available women of reproductive age (WRA) and children under five in the household. The Seca 874 U electronic scale (Hamburg, Germany) will be used for weight measurement, and measurements will be taken to the nearest 0.1 kg. Measurements will be taken twice, with a third reading taken if the difference between two weight measurements is greater than 50 grams in under-five children and greater than 500 grams in WRA. For infants and young children who cannot stand, weight will be measured using the indirect method, where the caregiver’s weight is first recorded, followed by the combined weight of the caregiver and the child. The child’s weight is then calculated by subtracting the caregiver’s from the combined weight. Recumbent length (children under 24 months of age) and standing height (children 24 months and above and mothers) will be measured using height boards (three slabs) to the nearest 0.1 cm. In the event of a discrepancy of more than 0.6 cm in children and 1 cm in WRA between the first two measurements, a third measurement shall be taken. Mid-upper arm circumference (MUAC) will be measured using a coloured MUAC tape to the nearest 0.1 cm in children under five. Measurements will be taken twice, with a third reading taken if the difference between the first two values is greater than 0.5 cm. A study supervisor will randomly visit the field sites to monitor the data collection. At the end of each day, the collected data will be transferred immediately from handheld devices to the AKU server via the internet.

### Statistical analysis

The collected data, stored on the server, will be exported to Stata V. 17 for analysis. Data will be cleaned under the guidance of the study investigators. Descriptive statistics will be presented: mean and SD or median with interquartile ranges for continuous variables, depending on the data distribution and frequency distributions for categorical variables. For all outcomes, including stunting and wasting z-scores, difference-in-difference (DID) analyses will be conducted, assuming parallel trends, that is, that intervention and control arms had parallel trends in the outcomes before the intervention was introduced. To address potential confounding, the DID will be adjusted for imbalanced factors at any time point. In addition, the model will also include covariates that are known to affect the outcome, including child factors (gender, age, morbidity status in the last 4 weeks, IYCF practices and vaccination status), maternal factors (obstetric history, history of previous pregnancies, dietary diversity, use of supplements and service utilisation of the Nashonuma Programme) and household factors (socioeconomic status, food security and water, sanitation and hygiene (WASH) indicators).

Sensitivity analyses will be conducted to assess the impact of including verus excluding the midline data, with the midline survey primarily serving to evaluate programme coverage and inform potential adjustments to the final analysis. A subgroup analysis will be performed to compare BNP and non-BNP recipients within the intervention districts. For child outcomes, an age-stratified analysis will be performed with adequate age groups to enable age-specific comparisons over time across study groups. All analyses will use robust standard errors to account for the clustering effect, and statistical significance will be evaluated at the 5% level with 95% CIs.

## Patient and public involvement

The study participants have not been involved in design, implementation or analysis. However, before the initiation of the study, we identified the community influencers/gatekeepers and conducted meetings with them to orient them about the overall goal and the activities of the study.

## Ethics and dissemination

Written informed consent will be obtained from all participants prior to enrolment in the study, emphasising their voluntary participation and right to withdraw at any time without consequence. There are no direct benefits to participants; however, they belong to the same population group receiving the intervention, and the study is evaluating its effectiveness in improving health outcomes for these vulnerable groups. Participation poses no risks to the individuals involved.

Measures will be implemented to protect the data using secure passwords and access restrictions limited to the immediate study personnel. All collected data will be handled with strict confidentiality and anonymised by removing personal identifiers during analysis, report writing and dissemination stages. The study has received approval from the Ethics Review Committee (ERC) of the Aga Khan University (ERC no: 2021-6412-18742) as well as the Pakistan National Bioethics Committee (NBC reference no: 4-87/NBC-761/22/1654). The study has been registered at clinicaltrials.gov as NCT06025786.

The evaluation of this national-level programme will assess its effectiveness in preventing stunting and improving the nutritional status of women and children. The results will provide critical evidence to support the integration of food-based interventions into Pakistan’s national health services infrastructure. The study findings will be shared widely through presentations at conferences, workshops and peer-reviewed journals. The findings will be made accessible to participants, local officials and healthcare facilities through community feedback sessions, tailored reports for district authorities and direct engagement with key stakeholders. Furthermore, materials will be translated into local languages and simplified for non-expert audiences to facilitate actionable knowledge. We will also provide policy briefs and reports to government and non-governmental organisations to inform future nutrition interventions in Pakistan and similar settings.
