# Supplementary material for: Impact assessment of Benazir Nashonuma Programme (BNP) on maternal, child health and nutritional status in Pakistan: a quasi-experimental study protocol
Source: BMJ Open. 2025 Jun 27;15(6):e094565. doi: 10.1136/bmjopen-2024-094565 (PMC12207155; doi:10.1136/bmjopen-2024-094565)
Supplement: Supplementary file 1 [file bmjopen-2024-094565.reviewer_comments.pdf]

PEER REVIEW HISTORY

BMJ Open publishes all reviews undertaken for accepted manuscripts. Reviewers are asked to complete a checklist review form and are provided with free text boxes to elaborate on their assessment. These free text comments are reproduced below.

ARTICLE DETAILS

Title (Provisional)

Impact Assessment of Benazir Nashonuma Program (BNP) on Maternal and Child Health & Nutritional Status in Pakistan: A Quasi Experimental Study Protocol

Authors

Muhammad , Shah; Malik, Asma Abdul; Soofi, Sajid; Habib, Atif; Umer, Muhammad; Rizvi, Arjumand; Ahmed, Imran; Leroy, Jef; Cousens, Simon; Bhutta, Zulfiqar Ahmed

---

VERSION 1 - REVIEW

**Reviewer**	**1**
**Name**	**Blackmore , Ivy**
**Affiliation**	**Washington University in St Louis**
**Date**	**20-Nov-2024**
**COI**	**None**

Abstract

• Line 36 – not clear why cross-sectional surveys are mentioned when it is a pre/post design.

• Lines 38-39 – the wording of this sentence is confusing, it sounds like the 13, 200 children will come from 734 households, which I don't think is what is intended

• Line 41 – observed changes or documented change?

• Line 49 – 13,200 households or 13,200 children (line 38)?

• Strengths and limitations – also risk of spillover

Intro

• Lines 91-93 – seems like this sentence should have a reference.

• Lines 111-171 – I'm not sure the detailed description of the intervention belongs in the Intro. Seems like it would be a better fit in the Methods. It would help to have a more concise description of the intervention.

Methods/Analysis

• Lines 178-179 – I feel like it would be clearer to “a quasi-experimental longitudinal design with data collection at baseline, midline, and endline in…”

• I am unclear on how there will be a baseline assessment for the intervention group as it sounds like the BNP intervention started in 2020. Are the study districts in places where they have yet to launch the intervention? Or maybe there is a way to describe this part of the intervention more clearly, possibly a flow diagram?

• Line 180 – what is/was the governments approach for selecting the intervention districts?

• Line 182 – Do you potentially mean the matching was done quantitatively? The indicators mentioned are quantitative.

• Study design – please describe specific study aims and hypotheses associated with those aims

• Eligibility criteria – any exclusion criteria? For example, index child is or becomes severely malnourished, participation in other nutrition programs, or declines to sign the informed consent?

• Outcomes and variables

o I suggest also tracking prevalence of diarrhea and other WASH/hygiene variables given the potential impact on nutritional status especially for children

o Any process evaluation outcomes to monitor and ensure consistent administration of the intervention (fidelity of delivery)?

• Sampling size/sampling strategy – it would help to have a figure of the proposed sampling strategy and/or the overall study design, it is hard to follow all the different parts and it would be useful to have a visual

o Any retention efforts?

o At what point will participants be considered lost to follow up and how will that be tracked?

o How will participant discontinuation be handled?

• Data collection

o Line 235 - back translating would help ensure accuracy not validity

o Line 239 - Describe how/where the data collectors will be recruited from and what their training will entail

o Line 243 – It is the household that is randomly selected not the child, correct?

• Line 275 – How will community gatekeepers be identified?

Statistical analysis

• Please expand on the description of potential covariates and efforts to reduce potential sources of bias

Ethics

• Describe any potential benefits and risks of participating in the study

Dissemination

• What efforts will be made to share findings with participants and local level officials, health centers etc?

Discussion and limitations

• These sections seem to be missing from the body of the paper

---

**Reviewer**	**2**
**Name**	**Shinde, Sachin**
**Affiliation**	**Harvard University**
**Date**	**30-Dec-2024**
**COI**	**None**

Thank you for the opportunity to review the manuscript, Impact Assessment of the Benazir Nashonuma Program (BNP) on Stunting Prevention Among Children Aged 0–59 Months in Pakistan: A Quasi-Experimental Study Protocol. This timely and important topic would be of significant interest to the journal's audience.

The review file contains two manuscript versions, with notable differences in the sample sizes and listed outcomes. I have based my review on the version that includes the abstract. I hope my comments will contribute to strengthening this manuscript further.

Abstract

• Please spell out "BISP" in the methods section of the abstract.

• The introduction states that the program targets mothers of children aged 0–23 months and children aged 6–24 months, whereas the methods section mentions that 13,200 children aged 0–59 months will be enrolled. Please provide a rationale for this discrepancy.

• Including details on the intervention's duration and the chronology of pre- and post-intervention surveys would enhance clarity.

Main Text

• Line 136: Clarify the term "FC workers."

• Line 115: This line states that the quarterly cash transfer is unconditional, but conditions for receiving the cash are described in Line 162. Please reconcile this inconsistency.

• Consider stating the objectives of the evaluation before describing the intervention, including the secondary outcomes in the objectives.

• As the intervention involves multiple components, presenting a conceptual framework explicitly outlining how the intervention is expected to achieve its desired outcomes would be beneficial. This should include key mechanisms and processes of change, particularly given the behavioral, household, and institutional-level secondary outcomes listed in Table 4.

• Provide prospective timelines for intervention implementation and evaluation, including potential dates and duration.

• Lines 193–196: The sentence mentions that households registered in BISP are eligible, while also stating eligibility regardless of enrollment in BNP. Please clarify. A clearer explanation of how BNP and BISP components are interlinked would be helpful, including within the conceptual framework.

• If the evaluation uses a pre- and post-intervention design, why include children covered by intervention activities during the pre-intervention assessment period?

Additional Comments

• Line 200: Please add a citation.

• If BNP is a health and nutrition initiative for pregnant and lactating women, what is the rationale for including the secondary outcome of changes in the prevalence of non-pregnant WRA?

• Table 4: Clarify whether all eight items will be used to measure food insecurity at the household level and specify the data sources (e.g., self-reported or other).

• Clarify whether clustering within clusters is accounted for in the sample size calculation.

• Line 214: The end-point assessment is stated to occur two years after baseline, but the sample size calculation assumes changes over a three-year period. Please reconcile this difference.

• The sample size calculation assumes equal numbers of villages per district and cluster sizes, which may not yield accurate estimates. How will this be addressed in districts with fewer villages than required?

• Line 222: Define "SSU."

• Line 225: Provide a rationale and citation for the sampling method used. Address potential issues, such as selecting the oldest eligible child rather than the youngest in cases where program benefits may be age-specific.

• Why is MUAC not listed as an outcome but mentioned in the data collection process?

• Midline assessments are mentioned in the study design but not described elsewhere, particularly in the statistical analysis section.

• Statistical Analysis:

o What is the basis for assuming parallel time trends?

o How will the parallel trend assumption be tested, and how will results be attributed if it is not met?

o How will BNP and non-BNP recipients be compared within intervention districts when only BNP-registered participants are recruited?

• Lines 274–275: Revise for clarity, depending on the study status.

• Lines 276–277: Clarify whether participants and villagers are expected to attend conferences, workshops, or read peer-reviewed journals.

• Line 282: Provide more detail.

• Consider including a section on the study's strengths and limitations before discussing its implications could enhance the manuscript.

General Suggestions

• The manuscript contains grammatical errors and would benefit from thorough editing.

• Will data collectors and analysts be blinded to intervention status?

• Clarify the study status and timelines. If the study is at an advanced stage of implementation or data collection, a detailed statistical analysis plan would be valuable for readers.

**Reviewer**	**3**
**Name**	**Batura, Neha**
**Affiliation**	**University College London, Institute for Global Health**
**Date**	**07-Jan-2025**
**COI**	**None**

Thank you for the opportunity to review this protocol. Some considerations for the authors to reflect on:

- It would be worthwhile to mention what the secondary outcomes of the evaluation would be.

- It would also be worthwhile to reflect on the fact that there are no control groups in this evaluation. What the implication of the chosen study design with regards to this? This should be reflected in the strengths and limitations section.

- If the hypothesis is that better nutrition and health education during pregnancy will lead to improved outcomes for newborns, it is surprising to see that birthweight is not among the list of secondary outcomes. Why is the change in the prevalence of low birth weight of newborns not included?

- The protocol only discuss is the evaluation of impact on the health outcomes. However it does not seek to address any questions around implementation challenges or successes, acceptability of the supplementations or the educational sessions, or the cost and cost effectiveness, and the affordability of the programme. These research objectives would be essential to future implementation of similar programmes.

VERSION 1 - AUTHOR RESPONSE

Reviewer: 1
Dr. Ivy Blackmore , Washington University in St Louis
Comments to the Author:
Abstract
• Line 36 – not clear why cross-sectional surveys are mentioned when it is a pre/post design.

Removed the "cross-sectional" part from line 36 and line 179. Updated the manuscript in light of the comment.

• Lines 38-39 – the wording of this sentence is confusing, it sounds like the 13, 200 children will come from 734 households, which I don't think is what is intended

Made the required changes in the manuscript.

• Line 41 – observed changes or documented change?

Corrected to documented changes.

• Line 49 – 13,200 households or 13,200 children (line 38)?

Corrected to children.

• Strengths and limitations – also risk of spillover

Updated the manuscript and included: "There is a risk of spillover effects, where households in control areas, especially in district bordering with intervention districts, may indirectly benefit from the intervention, potentially diluting the measured impact" to the 4th bullet point.

Intro
• Lines 91-93 – seems like this sentence should have a reference.

Added the citation: Vaivada, Tyler, Michelle F. Gaffey, and Zulfiqar A. Bhutta. "Promoting early child development with interventions in health and nutrition: a systematic review." *Pediatrics* 140.2 (2017).

• Lines 111-171 – I'm not sure the detailed description of the intervention belongs in the Intro. Seems like it would be a better fit in the Methods. It would help to have a more concise description of the intervention.

Noted. We have moved the description of the intervention to the methods section. However, a short description of the ongoing government intervention gives a good context to the study and best placed in the introduction section.

Methods/Analysis

• Lines 178-179 – I feel like it would be clearer to "a quasi-experimental longitudinal design with data collection at baseline, midline, and endline in…"

Thank you for your feedback. We have corrected accordingly.

• I am unclear on how there will be a baseline assessment for the intervention group as it sounds like the BNP intervention started in 2020. Are the study districts in places where they have yet to launch the intervention? Or maybe there is a way to describe this part of the intervention more clearly, possibly a flow diagram?

The intervention started in the initial few districts in 2020 and was gradually upscaled in other districts. The baseline assessment thus included some districts within the early stages of the intervention and others where the intervention was planned. We are able to stratify districts by duration of prior intervention, intensity and coverage assessed in the baseline survey.

• Line 180 – what is/was the governments approach for selecting the intervention districts?

Updated the manuscript for clarity and added: We engaged the government from the outset in selecting both intervention and control districts. The government also informed us of the decision to scale up the intervention nationally and assisted in identifying suitable districts for inclusion in the assessment. While the government selected the most affected districts for the intervention, the research team identified control districts, ensuring they were matched to the intervention districts within the same province.

• Line 182 – Do you potentially mean the matching was done quantitatively? The indicators mentioned are quantitative.

Corrected.

• Study design – please describe specific study aims and hypotheses associated with those aims

Added under the study design section of methods.

• Eligibility criteria – any exclusion criteria? For example, index child is or becomes severely malnourished, participation in other nutrition programs, or declines to sign the informed consent?

There were no exclusion criteria except refusal to consent.

• Outcomes and variables

o I suggest also tracking prevalence of diarrhea and other WASH/hygiene variables given the potential impact on nutritional status especially for children

o Any process evaluation outcomes to monitor and ensure consistent administration of the intervention (fidelity of delivery)?

The prevalence of under-five diarrhea and other common morbidities, along with WASH and sanitation variables, are being captured in the household section of the questionnaire. Added to the table of outcomes.

Yes, the questionnaire has a section that assesses compliance with the intervention and fidelity of delivery.

• Sampling size/sampling strategy – it would help to have a figure of the proposed sampling strategy and/or the overall study design, it is hard to follow all the different parts and it would be useful to have a visual

o Any retention efforts?

o At what point will participants be considered lost to follow up and how will that be tracked?

o How will participant discontinuation be handled?

These serial surveys will be conducted within the same districts but not necessarily within the same households, given the random selection of households using a computerized database. This is a population-based study, which will give us district level estimates rather than panel estimates specific for individuals which is being studied separately in a different study.

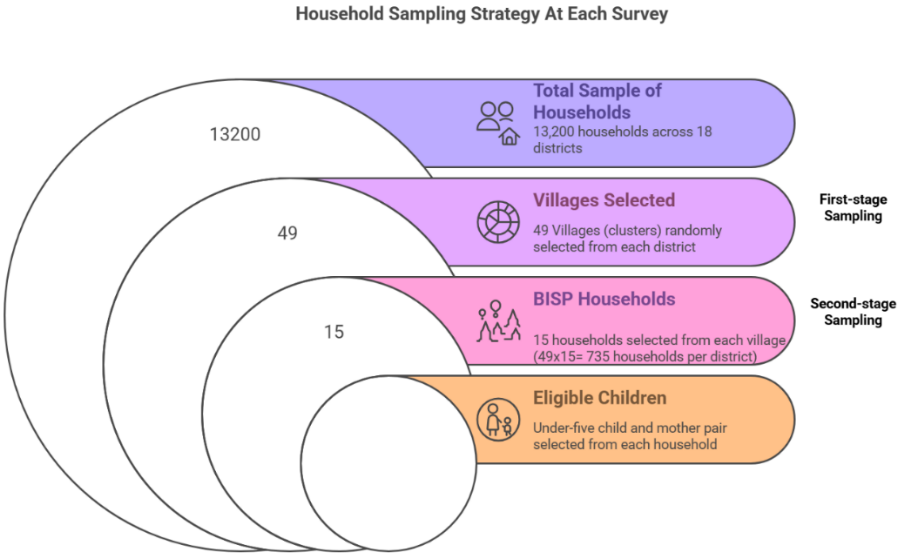

• Data collection

o Line 235 - back translating would help ensure accuracy not validity

Noted. And updated as suggested.

o Line 239 - Describe how/where the data collectors will be recruited from and what their training will entail

Added a paragraph under data collection section.

o Line 243 – It is the household that is randomly selected not the child, correct?

Yes, households. Corrected accordingly.

• Line 275 – How will community gatekeepers be identified?

We have revised this section and updated the relevant details under the ETHICS AND DISSEMINATION section.

Statistical analysis
• Please expand on the description of potential covariates and efforts to reduce potential sources of bias

Added under statistical analysis.

Ethics
• Describe any potential benefits and risks of participating in the study

Added this to the ethics part. "There are no direct benefits to participants; however, they belong to the same population group receiving the intervention, and the study is evaluating its effectiveness in improving health outcomes for these vulnerable strata. Participation poses no risks to the individuals involved."

Dissemination
• What efforts will be made to share findings with participants and local level officials, health centers etc?

Added "Findings will be made accessible to participants, local officials, and healthcare facilities through community feedback sessions, tailored reports for district authorities, and direct engagement with key stakeholders. Furthermore, materials will be translated into local languages and simplified for non-expert audiences to facilitate actionable knowledge."

Discussion and limitations
• These sections seem to be missing from the body of the paper

As per the requirements of the journal, limitations and strengths are presented right after the abstract. Discussion isn't there as it is a protocol manuscript.

Reviewer: 2
Dr. Sachin Shinde, Harvard University
Comments to the Author:
Thank you for the opportunity to review the manuscript, Impact Assessment of the Benazir

Nashonuma Program (BNP) on Stunting Prevention Among Children Aged 0–59 Months in Pakistan: A Quasi-Experimental Study Protocol. This timely and important topic would be of significant interest to the journal's audience.
The review file contains two manuscript versions, with notable differences in the sample sizes and listed outcomes. I have based my review on the version that includes the abstract. I hope my comments will contribute to strengthening this manuscript further.

Abstract
• Please spell out "BISP" in the methods section of the abstract.

Done. Thanks.

• The introduction states that the program targets mothers of children aged 0–23 months and children aged 6–24 months, whereas the methods section mentions that 13,200 children aged 0–59 months will be enrolled. Please provide a rationale for this discrepancy.

The surveys will be conducted at baseline, endline and endline over a period of three years. Children aged 0–23 months enrolled in BNP at baseline will age into the 24–59 months group over time (irrespective of whether they were part of the survey, as we are estimating district-level estimates rather than individual level). To assess the broader impact of the intervention, we aim to evaluate its effects on the overall nutritional status of children under five.

Including details on the intervention's duration and the chronology of pre- and post-intervention surveys would enhance clarity.

Noted with thanks. The duration of intervention has been updated in the methods section.

Main Text
• Line 136: Clarify the term "FC workers."

This is Facilitation center workers (FCWs). Spelled-out this this acronym in the text preceding line 136 for clarity.

• Line 115: This line states that the quarterly cash transfer is unconditional, but conditions for receiving the cash are described in Line 162. Please reconcile this inconsistency.

Line 115 refers to unconditional cash transfers (UCCTs) distributed by BISP to poor households. Meanwhile, Line 162 describes the conditionalities of the Nashonuma Program, a sub-program within BISP that specifically targets pregnant women and children aged 0–23 months. These beneficiaries belong to BISP households receiving the quarterly UCCTs but must meet additional conditions to receive Nashonuma benefits and cash (conditional cash transfer CCT).

• Consider stating the objectives of the evaluation before describing the intervention, including the secondary outcomes in the objectives.

We have added intervention details to the methods section after the objectives have been stated. Primary and secondary outcomes are stated in detail in table 4 in methods section.

• As the intervention involves multiple components, presenting a conceptual framework explicitly outlining how the intervention is expected to achieve its desired outcomes would be beneficial. This should include key mechanisms and processes of change, particularly given the behavioral, household, and institutional-level secondary outcomes listed in Table 4.

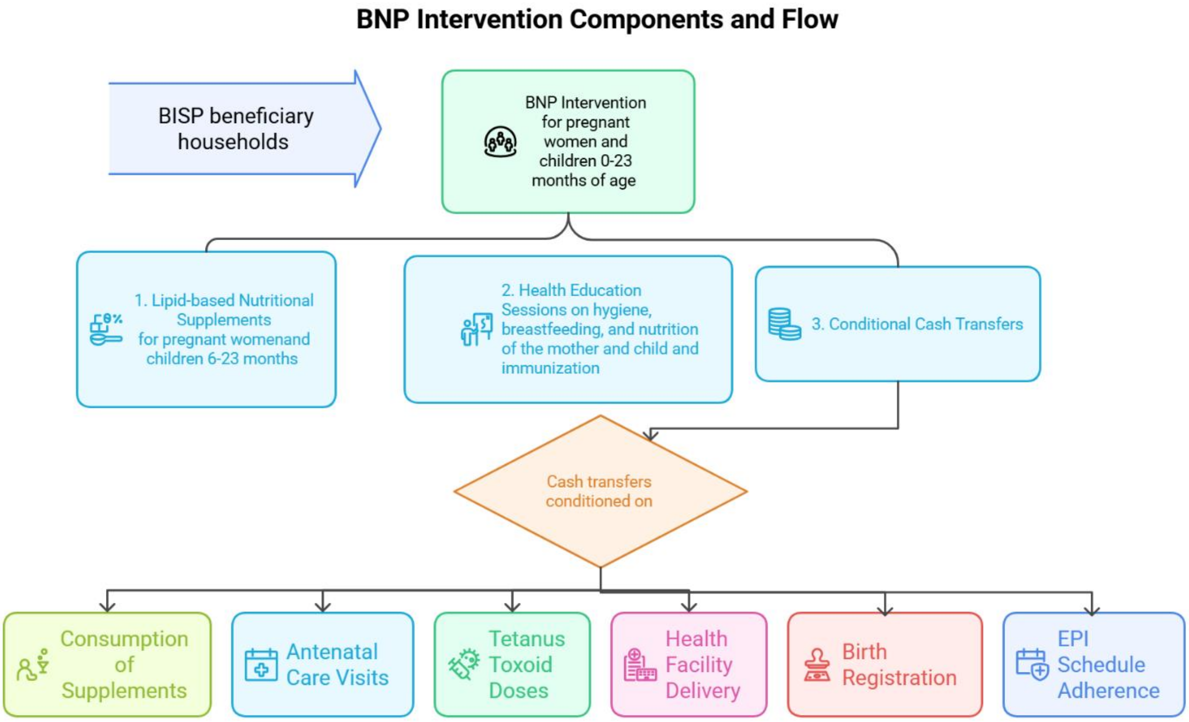

• Provide prospective timelines for intervention implementation and evaluation, including potential dates and duration.

Thanks for highlighting this. Timelines for the intervention are added in the methods section.

• Lines 193–196: The sentence mentions that households registered in BISP are eligible, while also stating eligibility regardless of enrollment in BNP. Please clarify. A clearer explanation of how BNP and BISP components are interlinked would be helpful, including within the conceptual framework.

The linkage between BNP and BISP is explained in detail in the intervention section.

• If the evaluation uses a pre- and post-intervention design, why include children covered by intervention activities during the pre-intervention assessment period?

Added. BNP enrolled children were included in the pre-intervention assessment to establish a baseline before program implementation. As the program had just been initiated, we assume it was the baseline status of the district BISP population.

Additional Comments

• Line 200: Please add a citation.

Added citation.

• If BNP is a health and nutrition initiative for pregnant and lactating women, what is the rationale for including the secondary outcome of changes in the prevalence of non-pregnant WRA?

We included non-pregnant women of reproductive age (WRA) because there is no standard BMI categories and cut-offs set for pregnant women, and BMI during pregnancy is not a reliable indicator of nutritional status. Assessing changes in BMI among non-pregnant WRA would provide a more accurate measure of the program’s broader impact on women's nutrition.

• Table 4: Clarify whether all eight items will be used to measure food insecurity at the household level and specify the data sources (e.g., self-reported or other).

Yes, we will cover all eight items, and it will be self-reported.

• Clarify whether clustering within clusters is accounted for in the sample size calculation.

Updated the analysis section.

Line 214: The end-point assessment is stated to occur two years after baseline, but the sample size calculation assumes changes over a three-year period. Please reconcile this difference.

Corrected. Thank you!

• The sample size calculation assumes equal numbers of villages per district and cluster sizes, which may not yield accurate estimates. How will this be addressed in districts with fewer villages than required?

The sample size was not distributed proportionate to the population because, at the time of the baseline survey, the intervention had only recently started in a few regions of Pakistan, such as Balochistan and Gilgit-Baltistan. As a result, a proportionate approach would have led to most of the sample being clustered in Punjab or a few regions of Sindh and Khyber Pakhtunkhwa. To ensure a more balanced representation, an equal distribution of the sample size was adopted.

We found no districts with fewer villages than required.

• Line 222: Define "SSU."

Thanks for highlighting this. Added and defined SSU, that is secondary sampling unit (which will be households).

• Line 225: Provide a rationale and citation for the sampling method used. Address potential issues, such as selecting the oldest eligible child rather than the youngest in cases where program benefits may be age specific.

Added citation and rationale and updated the relevant paragraph for clarity.

• Why is MUAC not listed as an outcome but mentioned in the data collection process?

Added to the list. Thanks for pointing out this.

• Midline assessments are mentioned in the study design but not described elsewhere, particularly in the statistical analysis section.

Updated the manuscript and added where relevant.

• Statistical Analysis:
o What is the basis for assuming parallel time trends?
o How will the parallel trend assumption be tested, and how will results be attributed if it is not met?
o How will BNP and non-BNP recipients be compared within intervention districts when only BNP-registered participants are recruited?

Explained in the statistical analysis section.

• Lines 274–275: Revise for clarity, depending on the study status.

We have revised the text and merged with the ETHICS AND DISSEMINATION section.

• Lines 276–277: Clarify whether participants and villagers are expected to attend conferences, workshops, or read peer-reviewed journals.

Updated the dissemination section.

• Line 282: Provide more detail.

Updated the details for data safety.

• Consider including a section on the study's strengths and limitations before discussing its implications could enhance the manuscript.

As per the requirements of the journal, the study's strength and limitations section has been added after the abstract in bullet points.

General Suggestions
• The manuscript contains grammatical errors and would benefit from thorough editing.

Thanks. We have mad ethe edits where required.

• Will data collectors and analysts be blinded to intervention status?

Data collectors and analysts are separate. The analysts will evaluate outcomes without allocating groups.

• Clarify the study status and timelines. If the study is at an advanced stage of implementation or data collection, a detailed statistical analysis plan would be valuable for readers.

Timelines added in the methods section. Also updated the analysis section.

Reviewer: 3
Dr. Neha Batura, University College London
Comments to the Author:
Thank you for the opportunity to review this protocol. Some considerations for the authors to reflect on:
- It would be worthwhile to mention what the secondary outcomes of the evaluation would be.

Table 4 describes secondary outcomes of the study.

- It would also be worthwhile to reflect on the fact that there are no control groups in this evaluation. What the implication of the chosen study design with regards to this? This should be reflected in the strengths and limitations section.

There are control districts instead of control groups or individuals. The comparison of estimates will be done between the intervention and control districts.

- If the hypothesis is that better nutrition and health education during pregnancy will lead to improved outcomes for newborns, it is surprising to see that birthweight is not among the list of secondary outcomes. Why is the change in the prevalence of low birth weight of newborns not included?

Birth weight is being studied separately in a cohort study that follows BNP-enrolled women through pregnancy with monthly follow-ups. Since this quasi-experimental study relies on population-level serial surveys rather than longitudinal follow-up of intervention households, capturing birth weight is not feasible.

- The protocol only discuss is the evaluation of impact on the health outcomes. However, it does not seek to address any questions around implementation challenges or successes, acceptability of the supplementations or the educational sessions, or the cost and cost effectiveness, and the affordability of the programme. These research objectives would be essential to future implementation of similar programmes.

The present protocol is for the Quasi Experimental Evaluation that assesses only the impact on health outcomes on district level. The implementation challenges, acceptability and cost effectiveness are part of another separate mixed-method evaluation.
